# Recurrent Spontaneous Coronary Artery Dissection

**DOI:** 10.1155/cric/3374538

**Published:** 2026-11-01

**Authors:** Harvey Nguyen, Paarth Sharma, Nicholas Berg, Saurav Uppal

**Affiliations:** ^1^ Internal Medicine Resident Department of Internal Medicine Summa Health Hospital Akron Ohio USA; ^2^ Cardiovascular Fellow Summa Health Hospital Akron Ohio USA; ^3^ Interventional Cardiology Summa Health Hospital Akron Ohio USA

## Abstract

Spontaneous coronary artery dissection (SCAD) is a rare but increasingly recognized cause of acute coronary syndrome (ACS) and sudden cardiac death. It has been associated with several predisposing factors, including hormone replacement therapy, autoimmune disorders, fibromuscular dysplasia (FMD), connective tissue diseases, atherosclerosis, migraine, pregnancy, and the postpartum period. SCAD most commonly affects young to middle‐aged women who present with acute myocardial infarction (MI) despite the absence of traditional atherosclerotic risk factors. Coronary angiography remains the gold standard for diagnosis, allowing visualization of the characteristic dissection patterns within the coronary vessel wall. Management is typically conservative as spontaneous healing of the dissection is common; however, revascularization with percutaneous coronary intervention (PCI) or coronary artery bypass grafting (CABG) may be necessary in patients with hemodynamic instability, ongoing ischemia, or failure of medical therapy.

## 1. Introduction

Spontaneous coronary artery dissection (SCAD) is a rare cause of acute coronary syndrome (ACS) and sudden cardiac death, predominantly affecting young, otherwise healthy women. The clinical presentation varies widely, ranging from asymptomatic cases to chest pain, ventricular arrhythmias, and sudden cardiac death, depending on the degree of coronary blood flow limitation. Although any coronary vessel can be involved, the left anterior descending (LAD) artery is the most affected vessel, involved in 32%–46% of all SCAD cases [1]. Long‐term studies have demonstrated recurrence rates as high as 11% within 10 years and 29% over 23 years [2]. Management strategies include conservative medical therapy, medical management with antiplatelet drugs, percutaneous coronary intervention (PCI), or coronary artery bypass grafting (CABG), depending on disease severity and hemodynamic stability. Here, we present a case of recurrent SCAD in a patient who is currently undergoing evaluation for an underlying connective tissue disorder.

## 2. Case Presentation

A female in her 60s with hypertension, hyperlipidemia, and a history of SCAD (2015) presented to the emergency department with acute chest pain. Her surgical history was notable for hysterectomy and oophorectomy, after which she discontinued hormone replacement therapy following her 2015 cardiac event. The patient′s symptoms began 2 days prior to presentation with a viral illness, nausea, and vomiting. On the morning of admission, she developed sudden‐onset, severe (10/10) chest discomfort while at work, which was accompanied by recurrent nausea and vomiting.

Laboratory evaluation revealed an elevated troponin level of 3214 ng/L. Electrocardiography (ECG) demonstrated ST‐segment elevation in leads I and aVL with reciprocal ST depression in leads II, III, and aVF (Figure 1), consistent with an acute lateral ST‐elevation myocardial infarction (STEMI). A STEMI alert was activated, and the patient was emergently taken to the cardiac catheterization laboratory.

**Figure 1:**
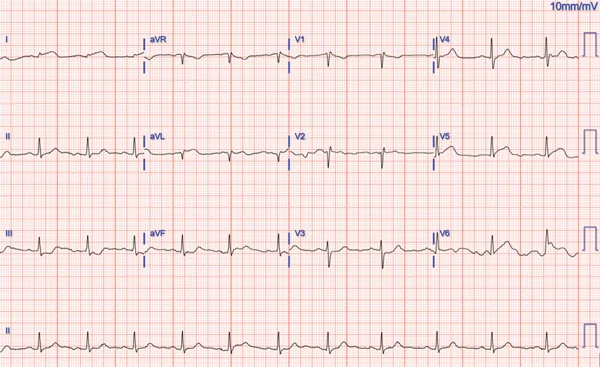
EKG of the patient on the day of admission, 2025, with ST elevation on leads I and aVL.

Coronary angiography revealed minimal disease in the native right coronary artery (RCA), circumflex artery, and LAD artery. No significant abnormalities were observed in the obtuse marginal branch (the site of her prior SCAD); however, there was evidence of diffuse narrowing in a small diagonal branch consistent with a spontaneous coronary dissection (Figure 2C). The prior dissection of the obtuse marginal branch and its subsequent healing are demonstrated in Figure 2A,B, respectively, whereas the previously normal diagonal branch is shown in Figure 2D.

**Figure 2:**
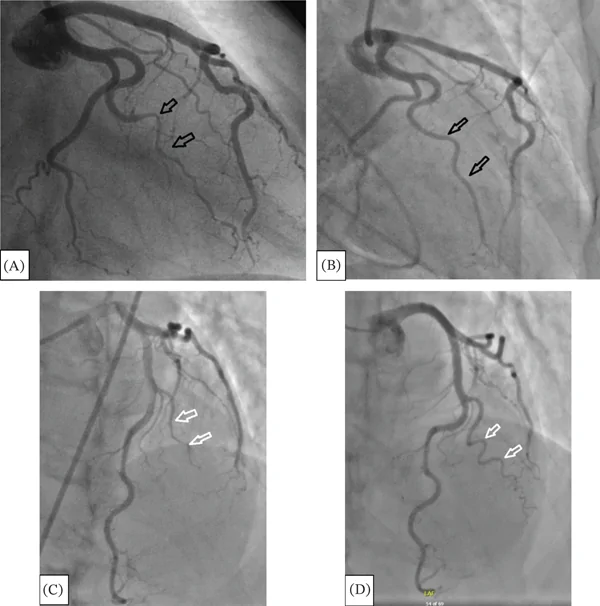
(A) Panel A showing spontaneous coronary dissection of obtuse marginal branch (black arrows) with acute coronary presentation in 2015. (B) Panel B showing healed dissection of the obtuse marginal branch (black arrows) on angiogram in 2025. (C) Panel C showing spontaneous coronary dissection of diagonal branch (white arrows) with acute coronary syndrome presentation in 2025. (D) Panel D showing the same diagonal branch without dissection (white arrows) previously captured during an angiogram in 2022.

The patient was admitted to the coronary care unit (CCU) and managed conservatively with nitroglycerin, a beta‐blocker, aspirin, and a statin. A postcatheterization transthoracic echocardiogram (TTE) demonstrated ejection fraction of 69% with no wall motion abnormalities. She was discharged 3 days later in stable condition.

Further evaluation for underlying etiologies included screening for fibromuscular dysplasia (FMD) with computed tomography (CT) angiogram of the head, chest, abdomen, and pelvis, which yielded unremarkable findings. Antinuclear antibody (ANA) testing was weakly positive. The patient is currently undergoing additional outpatient evaluation with rheumatology for a suspected connective tissue disorder.

## 3. Discussion

SCAD is a critical cause of ACS and sudden cardiac death. Although its precise pathophysiology remains unclear, several etiologies have been proposed, with underlying atherosclerosis being among the most frequently implicated factors. SCAD predominantly affects women and is typically diagnosed via invasive coronary angiography [3]. Management strategies vary depending on lesion characteristics and patient stability, ranging from conservative medical therapy to PCI or surgical revascularization.

Conservative medical therapies include antiplatelet therapy, beta blockers, statins, antianginals, and strict blood pressure control (target systolic blood pressure < 130 mmHg and heart rate 50–70 bpm). In this case, aspirin monotherapy was selected over dual antiplatelet therapy (DAPT) because the underlying pathology was driven by an intramural hematoma (IMH) and arterial wall disruption rather than atherothrombosis. Recent clinical guidelines favor aspirin monotherapy over DAPT in conservative management to mitigate the risk of increased IMH expansion [4].

SCAD most commonly occurs in young to middle‐aged healthy women. Predisposing factors include atherosclerosis, the peripartum period, underlying FMD, connective tissue and inflammatory disorders (e.g., Ehlers–Danlos and Marfan syndromes) [5], hormone replacement therapy, intense physical and emotional stressors, and the use of certain drugs, including oral contraceptives and cocaine [4]. Clinical presentation depends on the degree of coronary blood flow limitation and can range from asymptomatic cases to ACS, ventricular arrhythmias, or sudden cardiac death. Diagnosis is most often established via coronary angiography, and therapeutic decisions are guided by the extent and location of the dissection.

In this case, the patient had no signs, symptoms, or clinical features suggestive of a systemic connective tissue or inflammatory disorder, and her family history was unremarkable. Coronary angiography demonstrated a type 2 SCAD lesion in diagonal branch of the LAD system, which is also the most common type [1]. Her history of hormone replacement therapy during menopause is a suspected precipitating factor to her initial 2015 presentation [4], whereas the viral infection is potentially a predisposing factor for the recurrent event. The recurrence of SCAD may be associated with an underlying connective tissue disorder that is currently being investigated.

## 4. Conclusion

This case highlights the potential risk of recurrence in patients with SCAD and emphasizes the clinical importance of identifying predisposing factors, such as hormone replacement therapy or underlying connective tissue disorders, and the need for comprehensive vascular and rheumatologic evaluation. Conservative management remains the foundational strategy for hemodynamically stable patients, whereas invasive interventions should be reserved for ongoing ischemia or high‐risk lesions [6]. Long‐term follow‐up, risk factor optimization, and patient‐specific counseling are essential to minimize recurrence risks and improve long‐term clinical outcomes. This case underscores the need for heightened awareness of SCAD and individualized evaluation in patients presenting with recurrent events.

## Funding

No funding was received for this manuscript.

## Ethics Statement

The study complied with the ethical standards. IRB approval was not required for this case report.

## Consent

The authors confirm that written informed consent for the submission and publication of this case report, including images and associated text, has been obtained from the patient in accordance with journal policy. A copy of the written consent is available for review by the editor‐in‐chief of this journal on request.

## Conflicts of Interest

The authors declare no conflicts of interest.

## Data Availability

The data that support the findings of this study are available from the corresponding author upon reasonable request.
